# A case of alkaptonuria presenting with unexplained dark-stained diapers and spurious hyperoxaluria and proteinuria due to homogentisic acid interference

**DOI:** 10.11613/BM.2024.031002

**Published:** 2024-10-15

**Authors:** Thibault Vanhove, Margo Aertgeerts, Peter Witters, Daisy Rymen, Detlef Böckenhauer, Glynis Frans, Pieter Vermeersch

**Affiliations:** 1 Clinical Department of Laboratory Medicine, UZ Leuven, Leuven, Belgium; 2 Department of Paediatric Nephrology, UZ Leuven, Leuven, Belgium; 3 Department of Oncology, KU Leuven, Leuven, Belgium; 4 Centre for Metabolic Diseases, Department of Pediatrics, UZ Leuven, Leuven, Belgium; 5 Department of Development and Regeneration, KU Leuven, Leuven, Belgium; 6 Department of Cellular and Molecular Physiology, KU Leuven, Leuven, Belgium; 7 Department of Microbiology, Immunology and Transplantation, KU Leuven, Leuven, Belgium; 8 Department of Cardiovascular Sciences, KU Leuven, Leuven, Belgium

**Keywords:** preanalytical phase, interference, homogentisic acid, dark-stained urine, alkaptonuria, inborn errors of metabolism, case report

## Abstract

Alkaptonuria is characterized by the accumulation of homogentisic acid which causes dark coloration of urine upon standing, ochronosis, and arthritis. A 4-year old child was referred to our pediatric nephrologist with hyperoxaluria and a history of unexplained pink-to-brown discolouration of his diapers associated with a brown-staining of clothes and skin since he was six months old. He had no other symptoms and his past medical history only included minor child illnesses. His 11-month-old brother had the same dark discoloration of his diapers. Laboratory testing on a spot urine sample showed hyperoxaluria and nephrotic range proteinuria with low creatinine and normal albumin concentrations. Considered causes were hyperoxaluria, alkaptonuria, interfering substance, adulteration. The further diagnostic work-up revealed increased homogentisic acid in urine, compatible with alkaptonuria. Urinary creatinine and total protein measurements on Roche Cobas were, respectively, falsely decreased and increased in the presence of homogentisic acid. The false-low creatinine resulted in an elevated oxalate/creatinine ratio. Alkaptonuria can cause a false increase of results expressed *per* creatinine and should be excluded in case of an unexplained marked increase of urine total protein without a concomitant increase of albumin.

## Graphical abstract

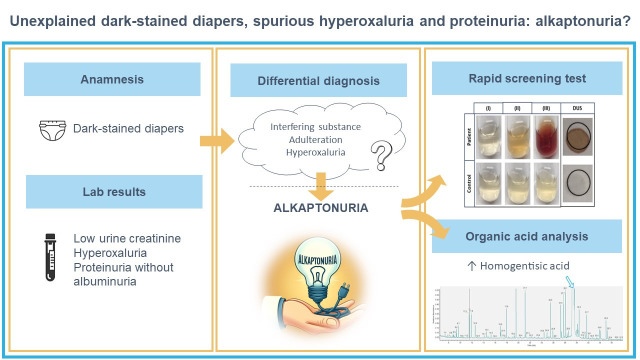

## Highlights

• Homogentisic acid (HGA) can cause a false increase of urinary results expressed *per* creatinine and a false increase of urine total protein measured with a method using benzethonium chloride

## Introduction

Alkaptonuria (AKU) is a rare autosomal recessive inborn error of the tyrosine metabolism caused by the deficiency of homogentisic dioxygenase (HGD) resulting in the accumulation of homogentisic acid (HGA) (*1*, *2*). The global prevalence is estimated at 1 *per* 250,000 to 1 000,000 with a higher prevalence in Slovakia and the Dominican Republic (*2*, *3*). The three major features of alkaptonuria are dark urine or urine that turns dark on standing (“black urine disease”), ochronosis (bluish-black pigmentation of connective tissue) and arthritis of the spine and larger joints (*2*). Ochronosis and arthritis typically only become symptomatic in adulthood (*2*, *4*). During childhood, patients remain largely asymptomatic which delays the diagnosis (*2*).

There is no curative treatment, but administration of nitisinone, a competitive inhibitor of 4-hydroxyphenylpyruvate dioxygenase, has been demonstrated to slow disease progression in adults (*5*, *6*) (*5*, *6*). Dietary protein restriction may result in a significantly lower HGA excretion and has shown some benefit in compliant children (*4*). People with AKU have a normal life expectancy, but quality of life is significantly impacted when patients eventually develop arthritis with joint pain and loss of movement (*2*).

The gold standard for the diagnosis of alkaptonuria is the detection of homogentisic acid in urine with gas chromatography-mass spectrometry (GC-MS) (*4*). Molecular genetic testing can identify homozygous or compound heterozygous genetic abnormalities in the HGD gene, that can help in family counselling (*2*, *4*). Medical imaging can help to assess the extent of joint involvement (computed tomography (CT) scan or magnetic resonance imaging), valvular abnormalities (2D-echocardiography) and calcification of coronary vessels (CT angiogram) (*4*). The broad range of differential diagnoses includes osteoarthritis, rheumatoid arthritis, ankylosing spondylitis, drug induced ochronosis, and porphyria (*2*, *4*).

## Case description

A 4-year old Caucasian boy was referred to our pediatric outpatient clinic because of hyperoxaluria discovered during a work-up for unexplained pink-to-brown discolouration of his diapers and brown-staining of clothes and skin. He was born at 38 weeks postmenstrual age.

Laboratory testing at the regional hospital before referral to our hospital showed moderate hyperoxaluria (up to 583 mmol/mol creatinine) and low urinary creatinine with Jaffé (0.97-2.30 mmol/L) on spot urine samples. The parents reported that the pink-to-brown urine stains were difficult to wash off and seemed almost bloody at times. He had no other symptoms. Total porphyrins in urine were normal. His younger brother, an 11-month-old infant, also showed dark discoloration of his diapers. Family history revealed that the maternal grandfather experienced 2 kidney stone episodes and the paternal uncle (father’s brother) experienced one kidney stone episode. Ultrasound of the patient’s kidneys, performed because of the hyperoxaluria, revealed some punctiform hyperreflections, suspicious for microlithiasis.

The anamnesis and clinical examination at our pediatric outpatient clinic after referral was unremarkable except for the diaper discolouration. Routine laboratory testing on a spot urine sample demonstrated nephrotic-range proteinuria (2754 mg/mmol creatinine) without significant albuminuria (10.2 mg/mmol creatinine), hyperoxaluria and an unexpectedly low creatinine concentration in the context of a concentrated urine (Table 1, first visit). Urine dipstick analysis for protein was negative. As these results were considered unlikely to be accurate, laboratory testing was repeated 6 weeks later on a 24-hour urine collection which confirmed proteinuria (1711 mg/mmol creatinine) without albuminuria and an unrealistically low creatinine excretion of 44.2 µmol/kg/d (normal range: 70-190 µmol/kg/d) (Table 1, second visit). The pediatric nephrologist contacted the laboratory medicine specialists to discuss the unexpected laboratory test results.

**Table 1:** Urine laboratory test results

**Laboratory test (unit)**	**First visit**	**Second visit** **(+ 6 weeks)**	**Third visit** **(+ 12 weeks)**	**Reference range**
Sample type	Spot urine	24-hour urine	24-hour urine	
Time (hh:mm)		24:00	24:00	
Amount (mL)		1050	900	
**Urine sample acidified**				
Creatinine (mmol/L)	0.81	0.62		NA*
Creatinine (mmol/24h)	NA	0.62		NA*
Oxalate (µmol/L)	327	101		
Oxalate (µmol/24h)		111		111-456
Oxalate (mmol/mol creat)	**402**	**162**		≤ 100
**Urine sample**				
Creatinine (mmol/L)	0.85	0.64	0.61	NA*
Creatinine (mmol/24h)		0.71	0.53	NA*
Total protein (mg/L)	**2340**	**1090**	**750**	≤ 150
Total protein (mg/24h)	NA	**1140**	**680**	≤ 150
Total protein (mg/mmol creat)	**2754**	**1711**	**1228**	≤ 17
Albumin (mg/L)	9	<3	5	≤ 20
Albumin (mg/24h)		<3	4	≤ 30
Albumin (mg/mmol creat)	10.2		8.0	NA
α1-microglobulin (mg/L)	< 4.0			≤ 12.5
Osmolality (mOsm/kg H_2_O)	1046	565		50-1200
Osmolality (mOsm/24h)		593		300-900
**Urinalysis**				
RBC count (/µL)	8	8	9	≤ 26
WBC count (/µL)	15	4	3	≤ 25
Manual microscopy	N	N	N	
pH	5.5	7.0	6.0	4.5-7.5
Relative density	1.029	1.013	1.015	1.005-1.030
WBC esterase	N	N	N	N
Nitrite	N	N	N	N
Protein	N	N	N	N
Glucose	N	N	N	N
Ketones	**P (2+)**	**P (2+)**	**P (2+)**	N
RBC Haem	N	N	N	N
Bilirubin	N	N	N	N
Creatinine (enzymatic), total protein and albumin were measured with Cobas c702 (Roche Diagnostics GmbH, Mannheim, Germany), oxalate with an enzymatic oxalate kit (Trinity Biotech Plc, Bray, Ireland), osmolality with OM-6050 (Arkray, Kyoto, Japan), urinalysis with UF-5000 and UC-3500 (Sysmex GmbH, Norderstedt, Germany), and α1-microglobulin with Immage 800 (Beckman Coulter, Brea, USA). *No reference intervals have been established for patients < 18 years old. Creat - creatinine. NA - Not available. N - Negative. P - Positive.				

Ethical approval for publication of this case report was obtained by the Ethical Committee of UZ Leuven (study number S68473). An informed consent form was signed by the father of the index patient.

## Laboratory analyses

The 24-hour urine collection was performed without any acidification. Upon arrival in the laboratory, the container was mixed and non-acidified aliquots were collected for all urine analytes except oxalate. Next, the remaining urine was acidified using 20% hydrochloric acid to achieve a pH value of 2 or lower, the container was mixed again, and the sample for oxalate was collected (*5*).

Urine chemistry tests were performed on a Roche Cobas 8000 ion-selective electrode (ISE) module and a c502 module (Roche Diagnostics GmbH, Mannheim, Germany). Meditape UC-10S dipstick (Eiken Chemical Co Ltd, Tokyo, Japan) analysis, urine particle analysis and digital microscopy were performed on an UC-3500, UF-5000 and UD-10 respectively (Sysmex GmbH, Norderstedt, Germany). Osmolality was measured using the osmometer OM-6050 (Arkray, Kyoto, Japan) and α1-microglobulin with Immage 800 (Beckman Coulter, Brea, USA). Urine protein electrophoresis and immunofixation were performed using the Hydrasys 2 (Sebia, Lisses, France). Oxalate testing was conducted enzymatically (Trinity Biotech Plc, Bray, Ireland).

Toxicology screening was carried out using Roche Cobas 8000 KIMS methods for drugs of abuse, and an in-house liquid chromatography with tandem mass spectrometry (LC-MS/MS) method on QTRAP 5500 LC-MS/MS (AB Sciex, Framingham, USA).

Urine organic acids was performed using an in-house method with gas chromatography with mass spectrometric detection (GC-MS, Trace GC with PolarisQ, Thermo Finnigan, Thermo Electron Corporation, Waltham, USA). Second tier testing for creatinine was performed with an in-house LC-MS/MS method (UPLC-MS/MS, Acquity UPLC I-Class/Xevo TQ-XS, Waters, Wexford, Ireland).

## Considered diagnoses and further investigations

Based on the proteinuria without albuminuria and low creatinine concentration, the laboratory medicine specialists considered interference by a drug or toxicological substance, a paraprotein or alkaptonuria as possible causes for the unexpected urine test results. Alkaptonuria causes an interference for total protein (benzethonium chloride) (falsely elevated), creatinine (enzymatic) and creatinine (Jaffé) (both falsely decreased) on Cobas, though not for albumin (immunoturbidimetric) in urine (*6*-*11*).

A new 24-hour urine collection for total protein, albumin, creatinine and oxalic acid was planned 12 weeks after the first visit to our outpatient clinic (Table 1, third visit). In addition, urine protein electrophoresis, urine organic acids analysis and urine toxicology testing were performed. Toxicological testing for administered or spiked substances as well as urine protein electrophoresis and immunofixation to exclude of Bence-Jones proteinuria were negative. Urine organic acid analysis revealed a large amount of HGA (9861 mmol/mol creatinine or 3.6 mmol/L) compatible with the diagnosis of alkaptonuria (> 1000 mmol/mol creatinine) (*12*). Measurement of creatinine using an in-house confirmation method (LC-MS/MS) confirmed that creatinine was false low due to interference by HGA (2.76 mmol/L compared to 0.61 mmol/L with creatinine (enzymatic) on Cobas) (*11*).

**Figure 2:**
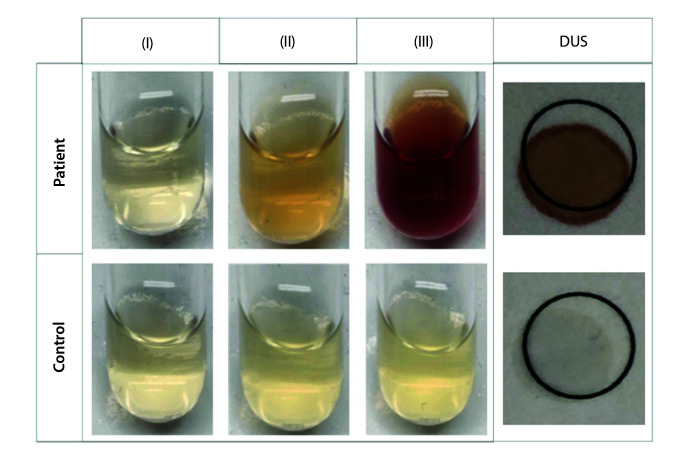
Alkalization of urine and dried urine paper spot (DUS). Accelerated oxidation (brown colour) of HGA by addition of alkaline solution (1M NaOH) to a patient sample and control sample. Alkalization of 0.8 mL patient urine sample and control sample with respectively 10 (I), 30 (II), 50 (III) µL 1M NaOH added and an incubation time of 3 minutes following each addition at room temperature (*15*). Alkalization of dried urine spots with 30 µL urine and 10 µL 1M NaOH added (*16*). HGA - homogentisic acid.

As urine organic acid analysis and creatinine with LC-MS/MS are not readily available 24/7, we also evaluated a rapid screening test. We performed the accelerated oxidation of HGA test by the addition of alkaline (1M NaOH) solution both on liquid urine and in a dried urine paper spot (DUS) and found with both methods a colour change indicative of the presence of HGA (Figure 2) (*13*, *14*). The incremental addition of NaOH (up to 50 µL) resulted in a dark brown coloration with a pH of 10 for the patient sample and 11 for the control sample. Between each addition there were 3 minutes of incubation at room temperature (Figure 2). We observed a colour change after the addition of 10 µL 1M NaOH to DUS, as described by Jacomelli *et al.* (*14*). In contrast, we needed 30 µL of 1M NaOH for the liquid urine sample before any notable colour change occurred while Tokuhara *et al.* reported a colour change after addition of only 10 µL (*13*). This might be due to the lower concentration of HGA in our child (3.6 mmol/L) compared to the 10 adult patients in the study of Jacomelli *et al.* (5.3-20.3 mmol/L) (*14*).

We noted an interference on the urine dipstick analysis (Sysmex Meditape UC-10S). The Sysmex UC-3500 automated dipstick reader gave an error code (“?”: Abnormal coloration) for ketones. When repeated manually, the dipstick showed indeed an aberrant colour reaction for ketones compared to the reference colour scale printed on the container. We considered interference by phenazopyridine, a urinary “analgesic” which can interfere with urinalysis tests that rely on colour reactions or spectrometry such as ketones due to its dye properties and because phenazopyridine can cause urine discoloration (reddish-orange) (*15*). The parents did, however, not report any medication use and phenazopyridine was taken of the Belgian market more than 1 year before the first visit to our outpatient clinic.

The younger brother, who was not yet potty trained when his older sibling was diagnosed, was tested at the age of 21 months. Organic acid analysis confirmed the suspected diagnosis of AKU (HGA 10,114 mmol/mol creatinine). The screening test on DUS was also positive in this patient.

## What happened?

Patients with alkaptonuria excrete large amounts of HGA in urine (> 1000 mmol/mol creatinine) (*12*). Oxidation of HGA excreted in the urine produces a pigmented polymer which causes the urine to turn dark over time upon standing or exposure to an alkaline agent. Darkening of the urine may, however, only occur several hours after voiding and many patients never observe any abnormal colour of their urine as was the case in our patient (*2*). Homogentisic acid did, however, cause brown discolouration of his diapers and brown-staining of clothes and skin.

The presence of HGA causes significant interference in a range of urine laboratory tests including creatinine (enzymatic), creatinine (Jaffé), total protein (benzethonium chloride) (*7*, *9*, *11*). The false decreased urine creatinine was the cause of the apparent hyperoxaluria, while the interference with the total protein assay caused the unexplained significant proteinuria without albuminuria. The low urine creatinine results (< 0.88 mmol/L) triggered automatic reruns in our laboratory to rule out a technical problem (*e.g.* air bubble) as such a result is unlikely, but these reruns gave similar results (0.85 and 0.85 mmol/L, 0.65 and 0.63 mmol/L, 0.61 and 0.58 mmol/L in the three separate samples). The unrealistically low creatinine excretion in the 24h urine collection together with the nephrotic range proteinuria without albuminuria and normal protein urinary dipstick raised the suspicion of interference by HGA. Other possible causes of interference that were considered were medication, adulteration, or a paraprotein. Further testing confirmed the presence of HGA and false-decreased urine creatinine and excluded the other causes of interference.

## Discussion

This case illustrates how interaction between clinicians and laboratory medicine professionals can solve a preanalytical mystery. The pediatric nephrologist was suspicious about the laboratory test results. The clinical laboratory performed an automated rerun because of the low creatinine results, but the reruns confirmed the initial findings. It was only when the pediatric nephrologist contacted the laboratory medicine professionals about the case, that alkaptonuria was included in the differential diagnosis.

Many patients with alkaptonuria are diagnosed during adulthood as children typically remain asymptomatic, but urine discoloration can be pathognomonic even in newborns (*2*, *16*, *17*). The only manifestation in this patient was the unexplained pink-to-brown discolouration of his diapers associated with a brown-staining of clothes and skin since he was six months old. No abnormal colour of the urine was noticed. This explains the diagnostic delay. It has estimated that only 21% of the children with AKU are diagnosed before the age of 1 year (*17*). This is reflected in our own patient population. Of the 7 AKU patients diagnosed in the last 15 years (including the two brothers), 3 were diagnosed as children (age 1-4 years old) and 4 as adults (age 33-48 years old). The HGA concentration at diagnosis varied between 8250 and 10,114 mmol/mol creatinine in the four children and between 1758 and 9613 mmol/mol creatinine in the four adult patients.

More than 250 HGD genetic variants have been reported in AKU patients. All variants identified in AKU patients worldwide are summarized in the HGD mutation database (http://hgddatabase.cvtisr.sk/) (*18*). A study of 172 AKU patients found no clinically relevant association between serum HGA or urine HGA excretion and residual HGD activity, indicating that dietary protein intake is more relevant for the HGA accumulation in the body than the genotype (*19*). The same study also did not find a clear association between genotype and clinical severity (*19*).

**Figure 1:**
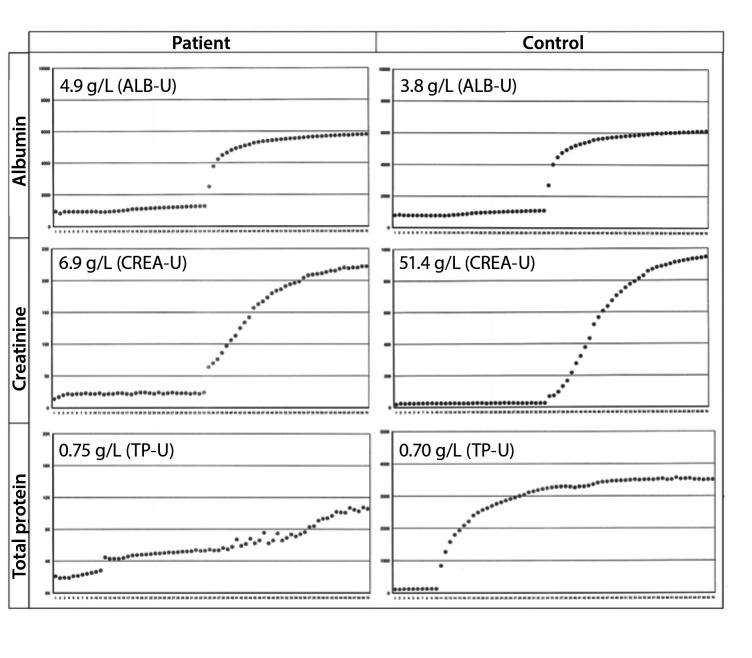
Reaction curves: Assay reaction response curves for albumin in urine (ALB-U), creatinine in urine (CREA-U), and total protein in urine (TP-U) measured on Cobas c502 (Roche Diagnostics GmbH, Mannheim, Germany). Left: patient sample. Right: control sample.

This case illustrates the difficulty of identifying patient samples with HGA interference despite the fact that the interference by HGA on urine and serum/plasma routine clinical chemistry assays is known and included in the product insert (*11*). The interference did not cause any technical flags on our Cobas analyzer. Nevertheless, a distinctly anomalous reaction pattern for creatinine and total protein was observed upon visual inspection, while the pattern was normal for albumin (Figure 1). Our automatic rerun trigger for urine samples with low creatinine values (< 10 mg/dL) could also not identify the problem as reruns gave similar results. We have discussed this problem with the manufacturer Roche Diagnostics who recognizes the problem, but indicated that they do not intend to change the technical flagging as this would be a significant change with regulatory implications. We therefore measure creatinine with LC-MS/MS when there are doubts about the accuracy of a urinary creatinine measurement and in patients with confirmed/suspected alkaptonuria.

Homogentisic acid interference can falsely lower urine creatinine (enzymatic) on Roche Cobas by more than 80%. While the creatinine (Jaffé) colorimetric method is less susceptible to interference by HGA, alkaptonuria can also cause false-low results with this method (*11*). The suspected mechanism for interference by HGA is an effect on the consumption of H_2_O_2_ for the enzymatic creatinine assay and a direct colorimetric effect for the urine for the Jaffé method (*7*, *11*). The benzethonium chloride method on Roche Cobas for urine total protein is also susceptible to significant interference by HGA as demonstrated by the 3 results of ≥ 750 mg/L in our patient (*7*). A discordance between significant proteinuria detected by a benzethonium chloride method *versus* a normal urinary protein dipstick results should alert the laboratory specialist of interference by HGA. Urine total protein methods using pyrogallol red, in contrast, appear not to be susceptible to interference by HGA (*7*, *9*). In retrospect, the patient had a normal result for urine total protein (40 mg/L) 15 months before the first visit to our hospital with an automated pyrogallol-based method (Beckman Coulter, Brea, USA) at the regional hospital. This further supports the observations that pyrogallol red-red based methods are not susceptible to HGA interference.

Curtis *et al.* studied the interference by HGA in Roche serum/plasma assays using a spiking experiment and found that HGA interference occurred predominantly with the peroxidase-coupled reactions creatinine (enzymatic), urate, total cholesterol, HDL cholesterol, triglycerides and to a lesser extend lactate (*7*). Of note, while Curtis *et al.* theoretically demonstrated that HGA concentrations of 100 µmol/L can cause a negative interference of up to 30% for plasma/serum creatinine (enzymatic) (*7*).

In conclusion, interference by HGA can cause a false increase of results expressed *per* creatinine and a false increase of urine total protein measured with a method using benzethonium chloride without a concomitant increase of albumin. Urine total protein methods using pyrogallol, in contrast, appear to be insensitive to interference by HGA. Alkaptonuria should be excluded in case of unexplained low creatinine and/or proteinuria without concomitant albuminuria. In patients with alkaptonuria urine creatinine should be measured using a method that is not susceptible to interference by HGA such as LC-MS/MS.

## What YOU should / can do in your laboratory to prevent such errors

Homogentisic acid can cause a false increase of urinary results expressed *per* creatinine and a false increase of urine total protein measured with a method using benzethonium chloride.
Alkaptonuria should be excluded in patients with unexplained high urine total protein without concomitant albuminuria.
In patients with alkaptonuria urine creatinine should be measured using a method that is not susceptible to interference by HGA, such as LC-MS/MS.
Laboratories can incorporate a rule in the laboratory information system to flag discordant results for urine total protein and albumin.
Suspected samples can be tested by adding alkaline solution to urine or dried spots to detect dark discoloration, or urine organic acid analysis.

## Data Availability

All data generated and analyzed in the presented study are included in this published article.
